# Outcome of treatment with trametinib in adults with histiocytic neoplasms in the United Kingdom

**DOI:** 10.1111/bjh.70617

**Published:** 2026-06-17

**Authors:** Rodothea Amerikanou, Talha Munir, Kristian Bowles, Christopher McNamara, Nikesh Chavda, Elizabeth Price, Jagdish Adiyodi, Alasdair Coles, Philippa Kelsey, Jeffery Smith, Richard Adams, Hayder Hussein, Rui Zhao, Trung Nguyen, Matthew Collin, Satyen Gohil

**Affiliations:** ^1^ University College London Hospitals NHS Foundation Trust London UK; ^2^ Haematology Department University College London London UK; ^3^ Leeds Teaching Hospitals Leeds UK; ^4^ Norwich Medical School University of East Anglia Norwich UK; ^5^ Norfolk and Norwich University Hospital Norwich UK; ^6^ University Hospitals Bristol NHS Foundation Trust Bristol UK; ^7^ Great Western Hospitals NHS Foundation Trust Swindon UK; ^8^ Royal Blackburn Teaching Hospital Blackburn UK; ^9^ Cambridge University Hospitals NHS Foundation Trust Cambridge UK; ^10^ Sheffield Teaching Hospitals NHS Foundation Trust Sheffield UK; ^11^ The Clatterbridge Cancer Centre NHS Foundation Trust Liverpool UK; ^12^ University Hospital of Wales Cardiff UK; ^13^ Queen Elizabeth Hospital University Hospitals Birmingham NHS Foundation Trust Birmingham UK; ^14^ Torbay Hospital, Torbay and South Devon NHS Foundation Trust Torbay UK; ^15^ Great Ormond Street Hospital for Children NHS Foundation Trust London UK; ^16^ Newcastle University Translational and Clinical Research Institute Newcastle Upon Tyne UK; ^17^ Newcastle Hospitals NHS Foundation Trust Newcastle Upon Tyne UK

**Keywords:** Erdheim–Chester disease, histiocytic sarcoma, Langerhans cell histiocytosis, Rosai–Dorfman disease

## Abstract

Trametinib (MEK inhibitor) shows efficacy in refractory and high‐risk adult histiocytic neoplasms. At a median follow‐up of 21.4 months, the clinical response rate was 81% (30/37) and the radiological response was 68% (25/37) (*n* = 37).

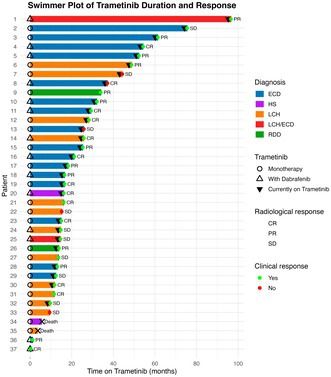

Histiocytic neoplasms are clonal inflammatory disorders of macrophages and dendritic cells that include Langerhans cell histiocytosis (LCH), Erdheim–Chester disease (ECD) and related disorders.^1^ They are commonly caused by activating somatic mutations in the Mitogen‐Activated Protein (MAP) kinase pathway, notably *BRAF* ^*V600E*^ and mutations of *MAP2K1*, which encodes Mitogen‐Activated Protein Kinase Kinase 1 (MEK1).^2^, ^3^, ^4^ Patients who do not respond to conventional chemotherapy or immunomodulatory treatment often respond to B‐Raf proto‐oncogene, serine/threonine kinase (BRAF) and MEK inhibitors, although efficacy can be limited by side effects.^5^, ^6^, ^7^, ^8^ MEK inhibitors have been used in tandem with BRAF inhibitors in patients with *BRAF* ^*V600E*^, and can also be used as monotherapy when alternative mutations are present.^3^, ^5^ The BRAF inhibitor vemurafenib is approved by the US Food and Drug Administration (FDA) and has orphan medicinal product designation in Europe.^9^ The MEK inhibitor cobimetinib is also approved in the United States for histiocytic neoplasms.^5^ Trametinib, an alternative MEK inhibitor, has been described in two adult studies, one describing 20 patients with ECD^10^ and another describing 26 non‐LCH patients^11^ but further evidence of its efficacy and tolerability in a range of histiocytic neoplasms is desirable.

In this multicentre retrospective study, we included patients with histiocytic neoplasms in the United Kingdom, who commenced trametinib at age ≥ 18 years, before 30 April 2024. We identified patients from the minutes of the UK Histiocytosis Advisory Panel (HAP) and collated data from 37 adults who received trametinib as monotherapy (*n* = 23) or in combination with the BRAF inhibitor dabrafenib (*n* = 14) (Figure 1, Table 1).

**FIGURE 1:**
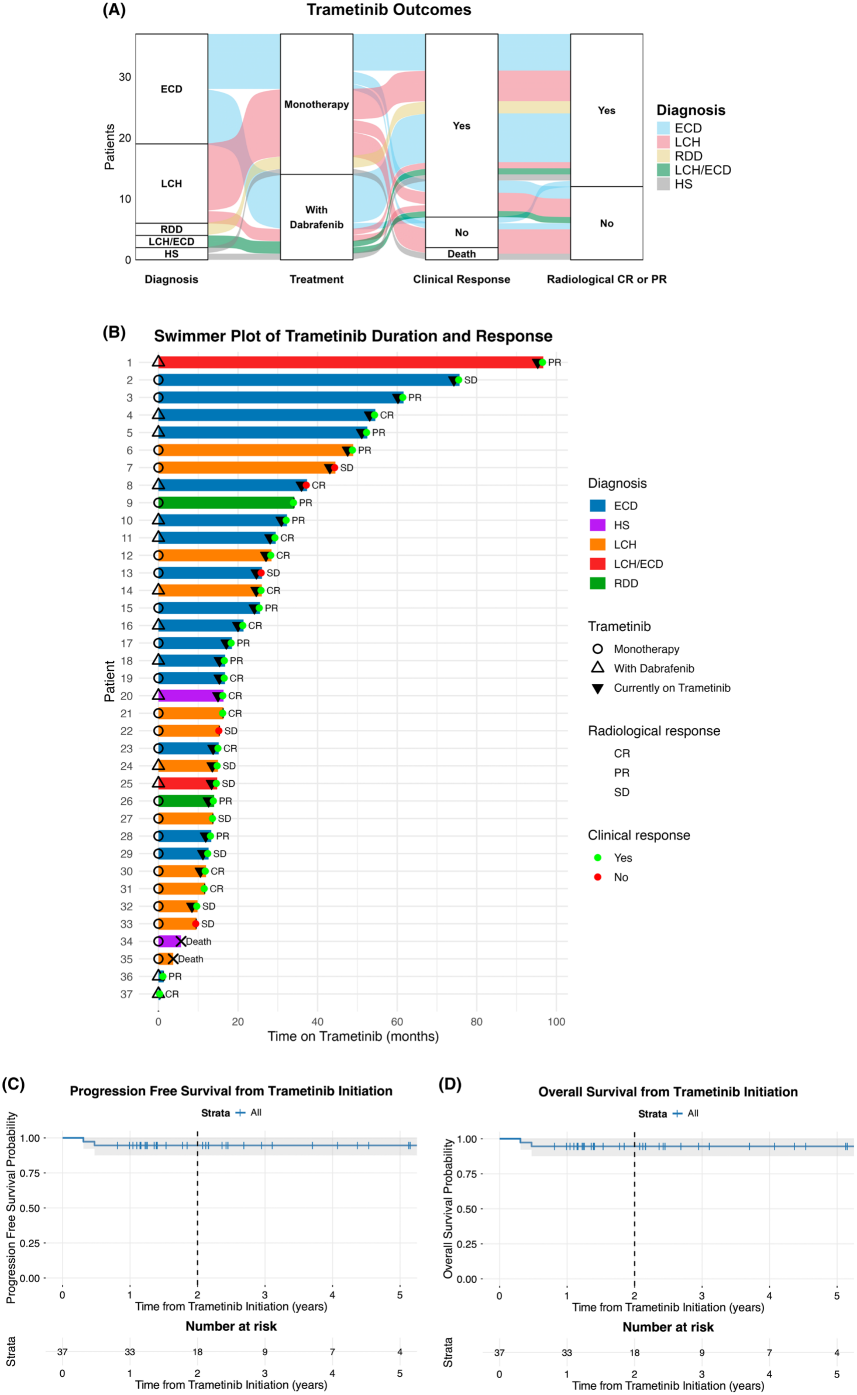
Response to therapy. (A) Alluvial plot describing diagnosis, treatment, clinical and radiological responses. Each alluvium represents a patient annotated as Erdheim–Chester disease (ECD) (blue), Langerhans cell histiocytosis (red), Rosai–Dorfman disease (RDD) (yellow), LCH/ECD (green) and histiocytic sarcoma (HS) (grey). (B) Swimmer plot of trametinib duration and response. Horizontal bars represent individual patients with bar length corresponding to treatment duration. Bar colours correspond to the patient diagnosis. Symbols at the left edge correspond to trametinib monotherapy (circle) or combination with dabrafenib (triangle). Symbols at the right‐hand side edge of the plot correspond to radiological response: complete response (CR), partial response (PR), stable disease (SD); clinical response (yes: green; red: no) and death (X). (C) Progression‐free survival (PFS) in years from trametinib initiation. Vertical line at 2 years marks the 2‐year PFS. (D) Overall survival (OS) in years from trametinib initiation. Vertical line at 2 years marks the 2‐year OS. [Colour figure can be viewed at wileyonlinelibrary.com]

**TABLE 1:** Patient characteristics split by treatment cohort (trametinib monotherapy and trametinib with dabrafenib).

Patient	Age at diagnosis and gender (y, sex)	diagnosis	Biopsy site	BRAF status	Other mutation	Sites involved at diagnosis	Prior lines of therapy	Endocrinopathy	Toxicity (G3/G4 only)	Reason for stopping trametinib	Clinical response	Radiological response
*On trametinib*												
1	22, M	ECD	Bone	WT	Nil	Bone Neurodegeneration—cerebellum	0				Yes	SD
2	54, M	ECD	Bone	WT	Nil	Bone, skin, lung, liver, cardiac, perivascular, pituitary	2	Vasopressin deficiency Hypopituitarism			Yes	PR
3	22, M	LCH	Lymph node	V487‐T491del	Nil	Lymph node, pituitary, hypothalamus, spleen, bone, skin	3	Vasopressin deficiency Hypopituitarism	Constipation G3		Yes	PR
4	1, M	LCH	GI	Unknown—poor specimen quality	Unknown—poor specimen quality	Bowel	2				No (ND)	SD
5	53, F	RDD	Dura	WT	Nil	Lymph node, dura, nerves	6		Myositis/raised CK G3	Corneal Oedema	Yes	PR
6	30, F	LCH	Lung	WT	Nil	Lung, bone, anal mucosa	2				Yes	CR
7	53, M	ECD	Bone	WT	Nil	Bone, peri‐renal, neurodegenerative—cerebellum, pons	0				No (ND and persistent bone disease)	SD
8	62, M	ECD	Peri‐renal	WT	MAP2K1	Peri‐aortic, peri‐renal, adrenal, bone, cardiac, skin—xanthelasma, pituitary	1	Hypopituitarism			Yes	PR
9	38, M	ECD	Skin	WT	Nil	Bone, cardiac, peri‐renal, dura, peri‐aortic, skin	2				Yes	PR
10	49, M	LCH	Bone	Unknown—poor specimen quality	Unknown—poor specimen quality	Bone, lung	4	Hypopituitarism	Pneumonitis G3 Myositis/raised CK G3	Pneumonitis	Yes	CR
11	47, M	ECD	Bone	Unknown	Unknown	Bone, peri‐renal, perivascular, pituitary	1	Vasopressin deficiency Hypopituitarism			Yes	CR
12	67, M	ECD	Peri‐renal	WT	MAP2K1	Cardiac, perivascular, peri‐renal, skin	0	Hypopituitarism	Rash G3		Yes	CR
13	14, M	LCH	Pituitary	Unknown	Unknown	Pituitary, cerebellum	2	Vasopressin deficiency		Compliance	No (ND)	SD
14	46, F	ECD	Retro‐orbital	V600E	Nil	Bone, retro‐orbital, perivascular, peri‐renal	2				Yes	PR
15	36, F	RDD	Lymph node and breast	WT	KRAS	Lymph node, bone, breast, orbital soft tissue	2				Yes	PR
16	49, F	LCH	Skin, lung and liver	Unknown	Unknown	Bone, liver, lung, skin	1	Vasopressin deficiency Hypopituitarism		Compliance	Yes	SD
17	64, M	ECD	Bone	WT	NRAS	Bone, paravertebral mass	5				Yes	SD
18	43, M	LCH	Skin	WT	Nil	Skin, lung, bones, liver	3	Vasopressin deficiency Hypopituitarism			Yes	CR
19	27, M	LCH	Lung	WT	Nil	Lung, skin, pituitary	0	Vasopressin deficiency			Yes	SD
20	1, M	LCH	Skin	WT	Nil	Skin	2	Hypopituitarism	Colitis G3	Colitis	No (ND)	SD
21	73, F	HS	GI	WT	Nil	Bowel, peritoneum	2		Rash G3	Disease‐related death (HS)	No (died)	PD
22	29, M	LCH	Lymph node	WT	Nil	Skin, lymph node, pituitary	3	Vasopressin deficiency Hypopituitarism		Patient preference (in CR)	Yes	CR
23	52, F	LCH	No biopsy (HRCT chest)	Unknown	Unknown	Lung, pineal cyst, cerebrum	0			Disease‐related death (pLCH)	No (died)	PD
*On trametinib with dabrafenib*												
24	62, F	ECD	Bone	V600E	Nil	Bone, pancreas, peri‐renal	2				Yes	PR
25	47, F	LCH/ECD	Skin	V600E	Nil	Lung, bone, cardiac, peri‐renal, pituitary, skin	3	Vasopressin deficiency Hypopituitarism			Yes	PR
26	53, M	ECD	Bone	V600E	Nil	Bone, peri‐renal, pituitary	2	Vasopressin deficiency			Yes	CR
27	48, F	ECD	Skin	V600E	Nil	Bone, skin, liver, lung, cardiac	3				Yes	PR
28	43, M	ECD	Bone	V600E	Nil	Bone Neurodegenerative	0				No (ND)	CR
29	51, M	ECD	Peri‐renal	V600E	Nil	Bone, peri‐renal, neurodegenerative—pons, cerebrum	0				Yes	CR
30	54, F	LCH	Bone	V600E	Nil	Skin, bone, pituitary	1	Vasopressin deficiency Hypopituitarism			Yes	CR
31	47, M	ECD	Brain	V600E	Nil	Cerebrum, pons hypothalamus	1	Vasopressin deficiency Hypopituitarism			Yes	CR
32	32, M	HS	Liver	D594G	Nil	Liver	1				Yes	CR
33	52, F	ECD	Skin	V600E	Nil	Bone, skin—xanthelasma	1	Vasopressin deficiency			Yes	PR
34	45, M	LCH/ECD	Pituitary	V600E	Nil	Pituitary, spinal epidural mass	0	Vasopressin deficiency Hypopituitarism			Yes	SD
35	53, M	LCH	Lung	V600E	Nil	Lung, neurodegenerative—cerebellum	0				Yes	SD
36	52, F	ECD	Bone	V600E	Nil	Bone, peri‐renal, perivascular, cardiac, omentum	2		Heart failure G3	Heart failure	Yes	PR
37	66, M	ECD	Orbital	V600E	Nil	Bone, orbit, perivascular, cardiac, pituitary, retroperitoneum	0		Hypertension G3	Uncontrolled hypertension	Yes	CR

Abbreviations: CK, creatine kinase; CR, complete response; ECD, Erdheim–Chester disease; F, female; G3, grade 3; G4, grade 4; GI, gastrointestinal, HRCT, high‐resolution computed tomography; HS, histiocytic sarcoma; KRAS, Kirsten rat sarcoma viral oncogene homolog; LCH, Langerhans cell histiocytosis; M, male; ND, neurodegenerative; NRAS, Neuroblastoma RAS viral oncogene homolog; PD, progressive disease, pLCH, pulmonary LCH, PR, partial response; RDD, Rosai–Dorfman disease; SD, stable disease; WT, wild‐type.

All diagnoses were reviewed by the HAP, according to clinical, radiological and pathological criteria. Mutation testing was performed by National Health Service Genomic Laboratory Hubs implementation of the Cancer Genomics panel M117 comprising next generation sequencing panel for histiocytic neoplasms, and Ribonucleic Acid (RNA) fusion panel using nucleic acids extracted from formalin‐fixed paraffin‐embedded (FFPE) biopsies as well as digital droplet polymerase chain reaction (PCR) for *BRAF* ^*V600E*^. Lesion genotype was reported at some level for 31/37 patients (Table 1). In six patients of unknown genotype, the original biopsy either failed to yield sufficient nucleic acid (*n* = 2) or was missing (*n* = 4). Mutations by histiocytosis subtype are shown in Figure S1a.

Patients had received a median of 2 lines of prior therapy (range 0–6). Further information regarding prior treatments is included in Figure S1b,c. Three patients had previously received a MAP kinase inhibitor (1 each of dabrafenib, vemurafenib or cobimetinib). Cobimetinib and vemurafenib were discontinued due to gastrointestinal (GI) bleeding and skin toxicity, respectively, and trametinib was added to the patient who had received dabrafenib due to lack of disease response. Trametinib was prescribed as first‐line treatment in 10/37 (27%); 5 as monotherapy and 5 in combination with dabrafenib. All were considered to have high‐risk disease; six had ECD including four with central nervous system (CNS) disease, three with LCH including two with advanced pulmonary involvement and two with CNS disease and one mixed LCH/ECD had a spinal canal lesion. The time from diagnosis to starting trametinib is shown in Figure S1d.

Trametinib and dabrafenib were provided by Novartis through a patient access scheme. The study was conducted as a clinical audit according to UK Health Research Authority guidelines. The cohort comprised 18 patients with ECD, 13 with LCH, 2 with mixed LCH/ECD, 2 with Rosai–Dorfman Disease (RDD) and 2 with histiocytic sarcoma. The median age at diagnosis was 48 years (range 1–73; Interquartile Range (IQR) 35–53); two patients had been diagnosed as children. There were 13 females (35%). Ethnicity data were reported for 30 patients, of which 25 were White (68%), 3 Asian, 1 Black and 1 mixed White‐Black ethnicity. The median Eastern Cooperative Oncology Group (ECOG) performance status was 1 (range 0–3).

Clinical response to trametinib was defined by an improvement in symptoms reported by the patient or clinician. Radiological response was assessed using Response Evaluation Criteria in Solid Tumors (RECIST) 1.1^12^ and Positron Emission Tomography Response Criteria in Solid Tumors (PERCIST)^13^ and categorised as complete response (CR), partial response (PR), stable disease (SD) or progressive disease (PD). PR and PD were not formally defined by radiological metrics in all cases. The disease response nearest the end of the study date was reported. Assessment of toxicity was determined by local physicians and dosing adjusted as necessary. Statistical analyses were conducted in R (v. 4.4.1) using base R, stats and epitools (v. 0.5.10.1). Kaplan–Meier curves were generated using the survminer (v. 0.5.0) package. Plots were designed with ggplot2 (v.4.0.1), ggalluvial (v. 0.12.5) and ggbeeswarm (v. 0.7.2) in RStudio. ChatGPT (OpenAI) was used as a tool for resolving R syntax errors.

At a median follow‐up of 21.4 months the clinical response rate was 81% (30/37) and the radiological response was 68% (25/37). The median overall survival and progression‐free survival were not reached and were both estimated at 95% at 2 years (Figure 1, Table 1). Two deaths occurred within 6 months, one in a patient with end‐stage pulmonary LCH and the other in one of two patients with histiocytic sarcoma; both were taking trametinib monotherapy. Lack of clinical response was observed in 26% (6/23) patients treated with trametinib monotherapy (2 deaths and 4 patients with neurodegenerative symptoms) compared with 7% (1/14) on trametinib and dabrafenib (neurodegenerative symptoms). Radiological non‐response was noted in 43% (10/23) treated with trametinib monotherapy compared with 14% (2/14) in the dual treatment group. Although non‐response was more frequent in the trametinib monotherapy group formal statistical comparison was not performed due to the small cohort size and event numbers.

Adverse events were observed in keeping with the known risks of trametinib. Most frequent was skin toxicity in 17/37 (46%), leading to treatment interruption in 10/37 (27%). GI adverse events were reported in 7/37 (19%) including nausea, abdominal pain, bleeding, constipation, a colitis flare and transaminitis. Raised creatine kinase occurred in 3/37 and arthralgia in 2/37. Infections in 2/37 required treatment interruption; one with urosepsis, respiratory infection and cellulitis and another with respiratory infection. Two patients had thrombotic events, one with pulmonary embolism and the other with basilar artery stroke. Hypertension was reported in 2/37, and acute kidney injury occurred once. Five patients discontinued trametinib owing to grade 3–4 toxicities including uncontrolled hypertension (ECD, dual treatment); cardiac failure (ECD, dual treatment); colitis flare (LCH, monotherapy with history of ulcerative colitis); corneal oedema (RDD, monotherapy); pneumonitis (LCH, monotherapy). Two further patients discontinued therapy owing to poor compliance and one by preference, having achieved CR. Of the 35 surviving patients, 27 remain on therapy at last follow‐up (77%).

This study is the largest of adults with histiocytic neoplasms treated with trametinib alone, or in combination with dabrafenib, and shows that it is an effective agent for histiocytic neoplasms. A recent study of non‐Langerhans cell histiocytic neoplasms in adults reported a similar response rate of 71% and 3‐year survival of 90%.^11^ Retrospective studies in children are also impressive with 100% favourable response in one study of 15 patients^14^ and 96% response in another 24 patients.^15^ Similar to most rare disease retrospective real‐world studies, our cohort is heterogeneous, with a small number of patients per disease group, and thus our conclusions may not be generalisable. However, our data add to the evidence demonstrating efficacy of trametinib and warrants dissemination to the medical community.^11^

In our study, the five patients who remained alive but did not respond had established neurodegeneration, highlighting the challenge posed by irreversible neurological damage. Earlier intervention with optimal CNS‐penetrating dosing may improve outcomes in neurodegenerative disease. Histiocytic sarcoma also presents a significant challenge: one patient died of progressive disease on trametinib, while the other remains alive on dual therapy with trametinib and dabrafenib.

Toxicity profiles are also similar, with rash the most prevalent, usually managed by dose interruption or reduction, topical steroids and antibiotics.^7^, ^11^ We report a high rate of clinical response but note that this was not based on a validated tool for collecting patient reported outcomes. In many patients, toxicity was managed by dose reduction, but we do not have information concerning initial and final drug doses. Further work is needed to identify the optimal dosing in histiocytic disorders and to determine the route to withdrawal of treatment after responses have been attained. Despite these limitations, this study provides important additional evidence of the utility of trametinib, alone or in combination with dabrafenib, for the management of histiocytic neoplasms.

## AUTHOR CONTRIBUTIONS

**Rodothea Amerikanou:** Conceptualization; writing – original draft; visualization; writing – review and editing; formal analysis; project administration; data curation. **Jagdish Adiyodi:** Writing – review and editing; data curation. **Christopher McNamara:** Writing – review and editing; data curation. **Hayder Hussein:** Writing – review and editing; data curation. **Talha Munir:** Writing – review and editing; data curation. **Philippa Kelsey:** Writing – review and editing; data curation. **Jeffery Smith:** Writing – review and editing; data curation. **Elizabeth Price:** Writing – review and editing; data curation. **Richard Adams:** Writing – review and editing; data curation. **Kristian Bowles:** Data curation; writing – review and editing. **Nikesh Chavda:** Writing – review and editing; data curation. **Alasdair Coles:** Writing – review and editing; data curation. **Matthew Collin:** Conceptualization; writing – original draft; visualization; writing – review and editing; formal analysis; data curation; supervision. **Rui Zhao:** Writing – review and editing; data curation. **Satyen Gohil:** Data curation; supervision; formal analysis; writing – review and editing; visualization; writing – original draft; conceptualization. **Trung Nguyen:** Writing – review and editing; data curation.

## FUNDING INFORMATION

RA's is supported by the CRUK City of London Centre Award (SEBCATP‐2022/100008). No other specific funding was received for this work. The funder had no role in study design, data collection and analysis, decision to publish or preparation of the manuscript.

## CONFLICT OF INTEREST STATEMENT

The authors declare no conflicts of interest.

## DISCLOSURE

The authors have nothing to disclose.

## ETHICS STATEMENT

No ethics committee approvals were required for this study.

## PATIENT CONSENT STATEMENT

No patient consent was required for this study.

## Supporting information


**Figure S1.** Cohort mutation and prior treatment characteristics.* (a) Mutations by histiocytosis subtype, (b) Non‐targeted treatments used prior to trametinib by the disease group: The barplot shows the number of patients receiving each treatment before the use of trametinib, grouped by diagnosis type. Bar height represents number of patients receiving each treatment. (c) Number of treatment lines across the different histiocytic neoplasms: The beeswarm plot shows the number of treatment lines received by each patient, grouped by disease type. Each point corresponds to a patient. (d) Delay in years of starting trametinib from time of diagnosis versus age at diagnosis: Each disease is represented by a different symbol and colour. AraC, cytarabine; CHOP, cyclophosphamide, vincristine, doxorubicin, prednisolone; ECD, Erdheim–Chester disease; HC, hydroxycarbamide; HS, histiocytic sarcoma; LCH, Langerhans cell histiocytosis; IFN‐α, interferon‐alpha; MMF, mycofenolate mofetil; MTX, methotrexate; RDD, Rosai–Dorfman disease. *ECD includes patients with LCH/ECD.

## Data Availability

De‐identified data supporting the study findings are available from the corresponding author, SG, upon reasonable request.
